# Amplification of poly(I:C)-induced interleukin-6 production in human bronchial epithelial cells by priming with interferon-γ

**DOI:** 10.1038/s41598-023-48422-9

**Published:** 2023-11-29

**Authors:** Norikazu Okuma, Masa-aki Ito, Tomoyoshi Shimizu, Atsuya Hasegawa, Shin’ya Ohmori, Kazuki Yoshida, Isao Matsuoka

**Affiliations:** 1 Laboratory of Pharmacology, Faculty of Pharmacy, Takasaki University of Health and Welfare, Takasaki-shi, Gunma 370-0033 Japan; 2 Department of Pharmacy, Japan Community Health Care Organization Gunma Chuo Hospital, Maebashi-shi, Gunma 371-0025 Japan; 3 Laboratory of Allergy, Faculty of Pharmacy, Takasaki University of Health and Welfare, Takasaki-shi, Gunma 370-0033 Japan

**Keywords:** Cell biology, Immunology, Molecular biology

## Abstract

Proinflammatory cytokine interleukin (IL)-6 was associated with disease severity in patients with COVID-19. The mechanism underlying the excessive IL-6 production by SARS-Cov-2 infection remains unclear. Respiratory viruses initially infect nasal or bronchial epithelial cells that produce various inflammatory mediators. Here, we show that pretreatment of human bronchial epithelial cells (NCl-H292) with interferon (IFN)-γ (10 ng/mL) markedly increased IL-6 production induced by the toll-like receptor (TLR) 3 agonist poly(I:C) (1 µg/mL) from 0.4 ± 0.1 to 4.1 ± 0.4 ng/mL (n = 3, *P* < 0.01). A similar effect was observed in human alveolar A549 and primary bronchial epithelial cells. TLR3 knockdown using siRNA in NCl-H292 cells diminished the priming effects of IFN-γ on poly(I:C)-induced IL-6 production. Furthermore, the Janus kinase (JAK) inhibitor tofacitinib (1 µM) inhibited IFN-γ-induced upregulation of TLR3, and suppressed poly(I:C)-induced IL-6 production. Quantitative chromatin immunoprecipitation revealed that IFN-γ stimulated histone modifications at the IL-6 gene locus. Finally, IFN-γ priming significantly increased lung IL-6 mRNA and protein levels in poly(I:C)-administrated mice. Thus, priming bronchial epithelial cells with IFN-γ increases poly(I:C)-induced IL-6 production via JAK-dependent TLR3 upregulation and chromatin remodeling at the IL-6 gene locus. These mechanisms may be involved in severe respiratory inflammation following infection with RNA viruses.

## Introduction

The outbreak of the coronavirus disease (COVID-19), an infection caused by severe acute respiratory syndrome coronavirus 2 (SARS-CoV-2), was recognized in late 2019 and has since spread rapidly worldwide^1^. On May 4th, 2023, the WHO declared the end of the state of emergency of international concern over the COVID-19 pandemic. However, many cases and deaths are still being reported from various countries^2^. Most patients with COVID-19 exhibit only mild symptoms such as fever, malaise, and dry cough. However, a certain percentage of patients, especially those with risk factors such as older age and diabetes, develop severe clinical symptoms^3^.

Patients with severe COVID-19 have been reported to have hyperinflammatory conditions caused by the excessive and uncontrolled release of proinflammatory cytokines such as interleukin-1 (IL-1), IL-2, IL-6, tumor necrosis factor (TNF)-α, interferon (IFN)-γ, IFN-inducing protein 10 kDa (IP10), and granulocytes-colony stimulating factor^4,5^. Among these, IL-6 levels have been shown to correlate with the severity of COVID-19 patient outcomes^5,6^. Excess IL-6 is involved in multi-organ inflammatory dysfunction^7^. IL-6 binds to the 80 kDa membrane-bound IL-6 receptor (IL-6R) and forms a functional complex with the signaling protein gp130, leading to downstream signaling and gene expression^8^. IL-6R is mainly expressed in immune-related cells but can be cleaved by proteinases and released into the blood as soluble IL-6R (sIL-6R)^9^. When sIL-6R binds to IL-6, it forms a complex with gp130, which is expressed both in immune and many other cell types, and induces inflammatory damage in various tissues^10^.

A retrospective study indicated that severe COVID-19 patients treated with tocilizumab, an anti-IL-6R monoclonal antibody, experienced clinical improvements, including fast defervescence, improved respiratory function, and discharged from the hospital^11^. Therefore, from the viewpoint of preventing aggravation in patients with severe COVID-19, revealing the mechanisms underlying excessively uncontrolled IL-6 production is important.

Various cell types are involved in IL-6 production in response to viral infection. Alveolar macrophages and recruited immune cells are considered a large source of inflammatory cytokines, including IL-6. In contrast, viruses targeting the respiratory tract first infect nasal or bronchial epithelial cells, which are known to produce various chemical mediators that affect the immune system^12^. Virus-derived double strand nucleic acids accumulated in epithelial cells are recognized by various innate immune sensors such as the Toll-like receptor (TLR) 3, TLR7, TLR8 and TLR9 that induce an induction of anti-viral type I and III IFNs, including IFN-α, β and λ^13,14^. In patients with severe COVID-19, the induction of these antiviral IFNs is reportedly lower than in mild patients^15–17^. In contrast, type II IFN-γ was found to be elevated with other inflammatory cytokines in patients with severe COVID-19^16,17^. IFN-γ is known to have priming effects that enhance lipopolysaccharide (LPS)-stimulated cytokine production by macrophage^18,19^. However, the role of IFN-γ on IL-6 production in the bronchial epithelial cells in response to viral infection remains to be investigated. In this study, we focused on human bronchial epithelial cells, which are more abundant than immune cells in lung tissue, and investigated the effects of IFN-γ on IL-6 production in response to the TLR3 agonist poly(I:C).

## Results

### Effects of IFN-γ on poly(I:C)-induced IL-6 production in NCI-H292 cells

**Figure 1:**
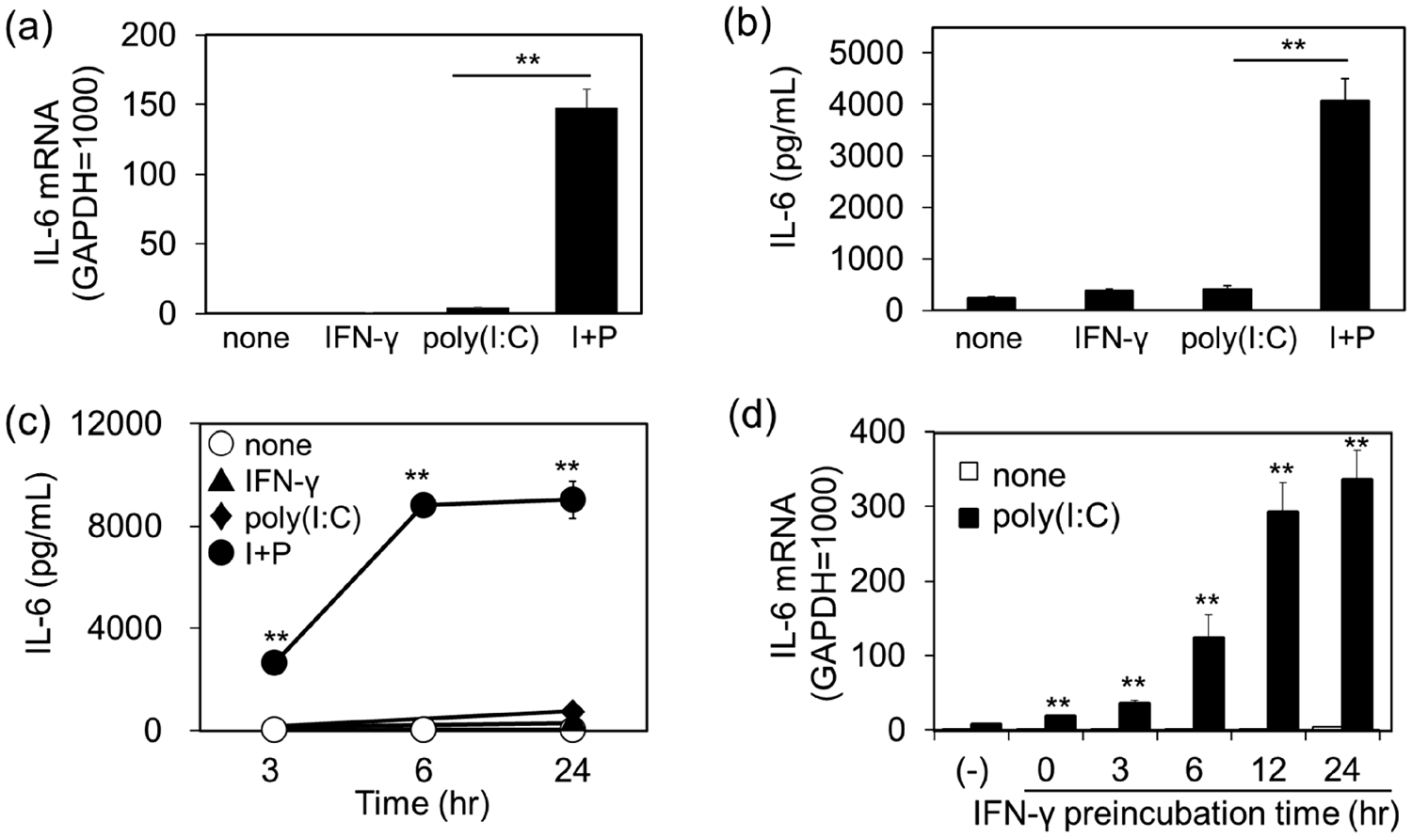
Amplification of poly(I:C)-induced IL-6 production by IFN-γ pretreatment in NCl-H292 cells. Cells were pretreated with IFN-γ (10 ng/mL) for 12 h, followed by stimulation with vehicle (none) or poly(I:C) (1 µg/mL) for 3 h for mRNA (**a**) and 6 h for ELISA (**b**) analyses. I + P indicates stimulation with IFN-γ and poly(I:C). IL-6 mRNA expression was examined by quantitative RT-PCR. Data were normalized to GAPDH mRNA levels. Values are shown as mean ± SEM (n = 3). **P < 0.01 versus the response to poly(I:C). The levels of IL-6 in the reaction medium were measured by ELISA. Values are shown as mean ± SEM (n = 3). (**c**) Cells were pretreated with IFN-γ 12 h, followed by stimulation with poly(I:C) (1 µg/mL) for 3, 6, and 24 h and the reaction medium was collected to measure IL-6 levels by ELISA. Values are shown as mean ± SEM (n = 3). **P < 0.01 versus the response to poly(I:C). (**d**) The cells were pretreated with IFN-γ (10 ng/mL) for different time periods and stimulated with poly(I:C) (1 µg/mL) for 3 h. IL-6 mRNA expression was examined using quantitative RT-PCR. Data were normalized to GAPDH mRNA levels. Values are shown as mean ± SEM (n = 3). **P < 0.01 versus the response to poly(I:C) alone.

In NCI-H292 cells, poly(I:C) (1 μg/mL) alone had little effect on IL-6 gene expression. Pretreatment with IFN-γ (10 ng/mL) for 24 h did not affect IL-6 gene expression. In contrast, poly(I:C) markedly increased IL-6 mRNA levels in IFN-γ-pretreated cells (Fig. 1a). In addition to these changes in gene expression, a synergistic increase in IL-6 protein production was observed after stimulation with poly(I:C) in IFN-γ-pretreated cells in a time-dependent manner, reaching a peak at 6 h of stimulation (Fig. 1b,c). The effect of IFN-γ on poly(I:C)-induced IL-6 mRNA expression increased with increasing incubation time. Compared to co-stimulation with IFN-γ and poly(I:C), poly(I:C)-induced IL-6 mRNA induction was increased markedly as a function of IFN-γ exposure time, reaching a maximum after 12 h (Fig. 1d). Based on these results, IFN-γ pretreatment lasted for 12 h in subsequent experiments.

**Figure 2:**
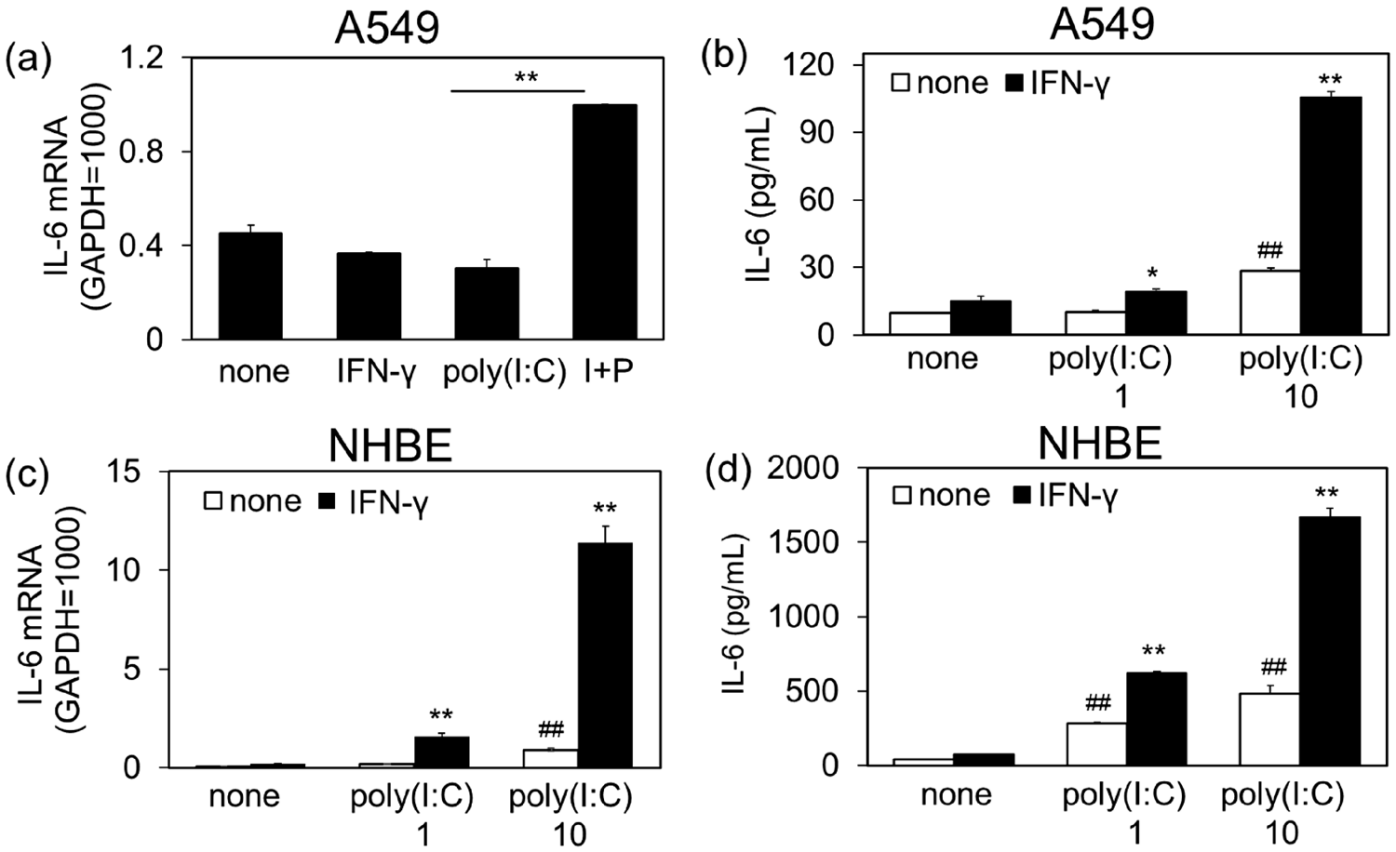
Amplification of poly(I:C)-induced IL-6 production by IFN-γ pretreatment in A549 (**a**,**b**) and NHBE (**c**,**d**) cells. Human alveolar epithelial cells A549 were pretreated with IFN-γ for 12 h, followed by stimulation with vehicle (none) or poly(I:C) (1 and 10 µg/mL) for 3 h for mRNA (**a**) and 6 h for ELISA analyses (**b**). (**c**,**d**) Normal human primary bronchial epithelial cells NHBE were also treated with IFN-γ and poly(I:C) similarly to A549 cells. IL-6 mRNA expression was examined by quantitative RT-PCR (**a**,**c**). Data were normalized to GAPDH mRNA levels. Values are obtained from three different experiments with cells from same doner and shown as mean ± SEM (n = 3). **P < 0.01 versus the response without IFN-γ. ^##^*P* < 0.01 versus none. Similar results with NHBE cells from another donor were obtained. The levels of IL-6 in the reaction medium were measured by ELISA (**b**,**d**). Values are shown as mean ± SEM (n = 3). **P < 0.01 versus the response without IFN-γ. ^##^*P* < 0.01 versus the response of none.

Next, we investigated the effect of IFN-γ on poly(I:C)-induced IL-6 production in different respiratory epithelial cells using the human alveolar epithelial cell line A549 cells and primary cultured human bronchial epithelial cells (NHBE). Although the magnitude and sensitivity of the response to poly(I:C) differed depending on cell type, poly(I:C)-induced IL-6 mRNA expression and protein secretion were significantly increased by the priming with IFN-γ in both A549 (Fig. 2a,b) and NHBE (Fig. 2c,d). Since the response was most pronounced in NCI-H292 cells, the following experiments were performed in NCI-H292 cells to investigate the mechanism underlying the priming effects of IFN-γ on poly(I:C)-induced IL-6 production.

### Effects of IFN-γ on poly(I:C)-induced IL-6 production and TLR3 expression

**Figure 3:**
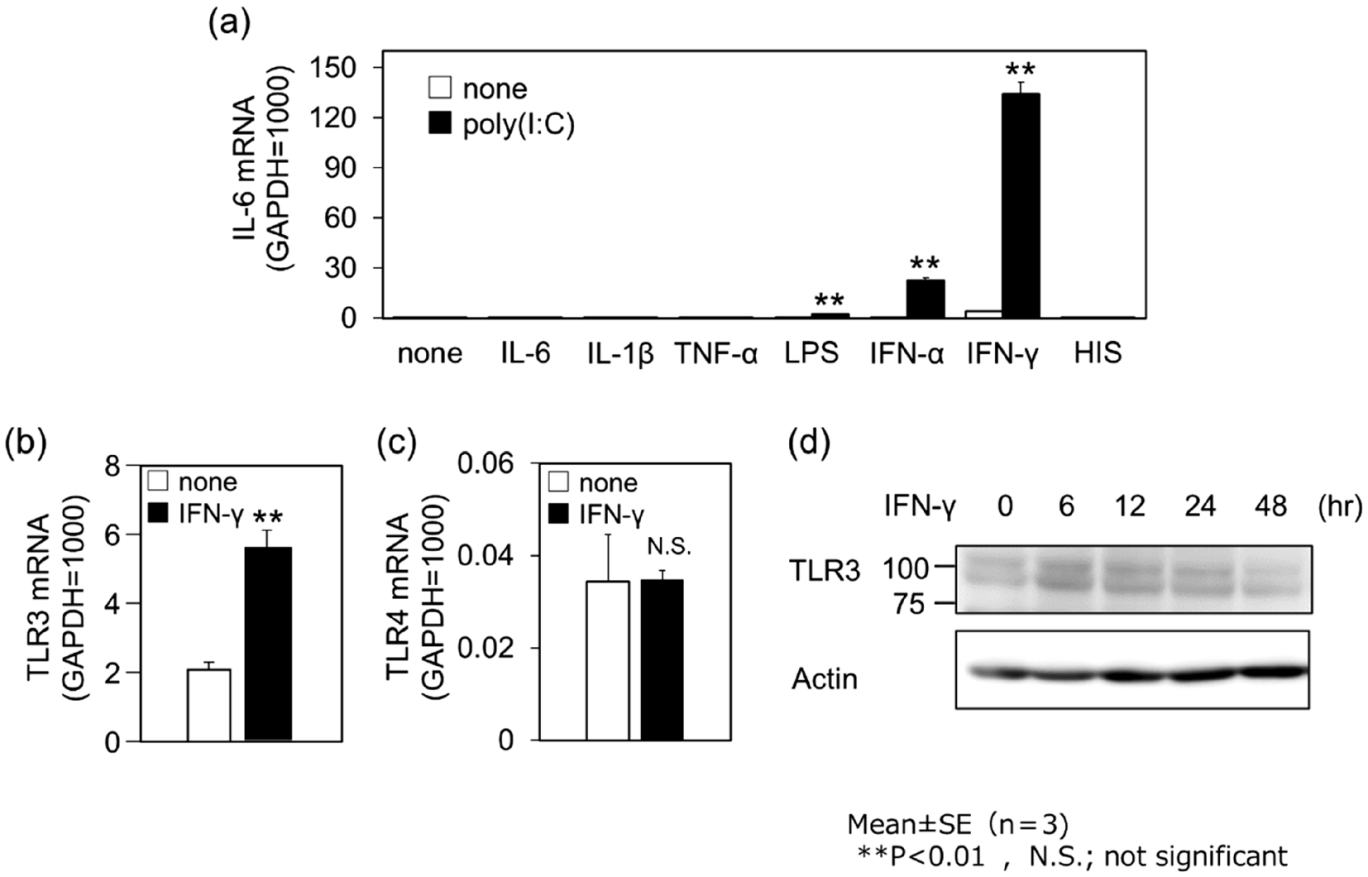
Stimulation with IFN-γ caused increase in poly(I:C)-induced IL-6 production and TLR3 expression. (**a**) Cells were pretreated with vehicle (none), IL-6 (10 ng/mL), IL-1β (10 ng/mL), TNF-α (10 ng/mL), LPS (1 µg/mL), IFN-α (10 ng/mL), IFN-γ (10 ng/mL), or histamine (HIS, 100 µM) for 12 h, and then stimulated with vehicle (open column) or poly(I:C) (1 µg/mL, black column) for 3 h. IL-6 mRNA expression was examined by quantitative RT-PCR. Data were normalized to GAPDH mRNA levels. Values are shown as mean ± SEM (n = 3). **P < 0.01 versus the response to none. Cells were stimulated with vehicle (none) or IFN-γ (10 ng/mL) for 12 h. TLR3 (**b**) or TLR4 (**c**) mRNA expression was examined by quantitative RT-PCR. Data were normalized to GAPDH mRNA levels. Values are shown as mean ± SEM (n = 3). **P < 0.01 versus the response to none. N.S., not significant. (**d**) Cells were stimulated with IFN-γ (10 ng/mL) for 0, 6, 12, 24, and 48 h. Cell lysates were subjected to western blot analysis for TLR3. Result shown is representative image.

We examined the effects of various cytokines on poly(I:C)-induced IL-6 expression. Although LPS and IFN-α slightly increased poly(I:C)-induced IL-6 mRNA expression, the effect was much smaller than that observed with IFN-γ (Fig. 3a). The other inflammatory cytokines, such as IL-6, IL-1β, and TNFα as well as histamine, which is known to potentiate the action of poly(I:C) in bronchial epithelial cells^19,20^, had no effect on poly(I:C)-induced IL-6 production (Fig. 3a). Poly(I:C) stimulates the endosomal double-stranded RNA (dsRNA) sensor TLR3^12^. We investigated the effect of IFN-γ on TLR3 expression in NCI-H292 cells. Priming with IFN-γ for 12 h increased TLR3 mRNA levels (Fig. 3b) without affecting TLR4 mRNA levels (Fig. 3c). Consistently, treatment of NCI-H292 cells with IFN-γ increased TLR3 protein levels to a maximum at 6 h, which lasted up to 24 h (Fig. 3d).

### Role of upregulation of TLR3 in priming effects of IFN-γ on enhanced IL-6 production induced by poly(I:C)

**Figure 4:**
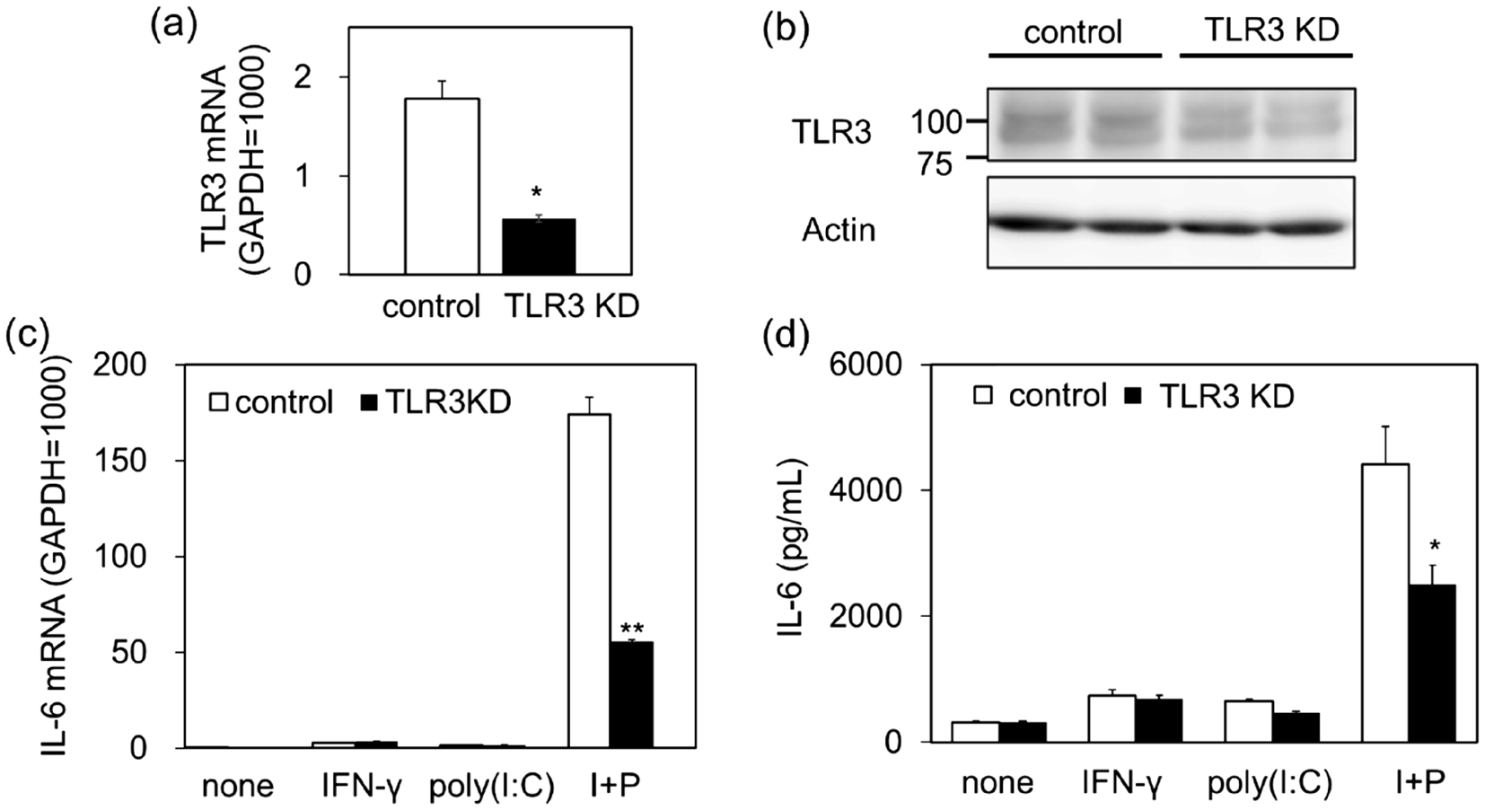
Effects of TLR3 knockdown on IL-6 production induced by stimulation with IFN-γ and poly(I:C). Cells were transfected with 50 pmol/well of human TLR3 gene-specific siRNA or negative control siRNA. After 2 d of culture, cell lysates were subjected to quantitative RT-PCR (**a**) (n = 3) and western blot analysis for TLR3 (**b**) (n = 2). (**c**) Cells were transfected with control or TLR3 siRNA. One day after transfection, cells were pretreated with IFN-γ (10 ng/mL) for 12 h, followed by stimulation with vehicle (none) or poly(I:C) (1 µg/mL) for 3 h. I + P indicates poly(I:C) stimulation in IFN-γ-treated cells. IL-6 mRNA expression was examined by quantitative RT-PCR. Data were normalized to GAPDH mRNA levels. Values are shown as mean ± SEM (n = 3). (**d**) Cells were stimulated as in (**c**) for 6 h. IL-6 levels in the reaction medium were measured by ELISA. Values are shown as mean ± SEM (n = 3). *P < 0.05 and **P < 0.01 versus the response to control.

To evaluate the role of TLR3 in the priming effects of IFN-γ on poly(I:C)-induced IL-6 production, we examined the effects of TLR3 knockdown using siRNA. First, we confirmed that the transfection of TLR3 siRNA into NCI-H292 cells decreased TLR3 mRNA and protein levels (Fig. 4a). In TLR3 siRNA-transfected NCI-H292 cells, the priming effects of IFN-γ on poly(I:C)-induced IL-6 mRNA elevation (Fig. 4b) and IL-6 secretion (Fig. 4c) were significantly inhibited.

### Involvement of Janus kinase (JAK) as a downstream signal of IFN-γ stimulation

**Figure 5:**
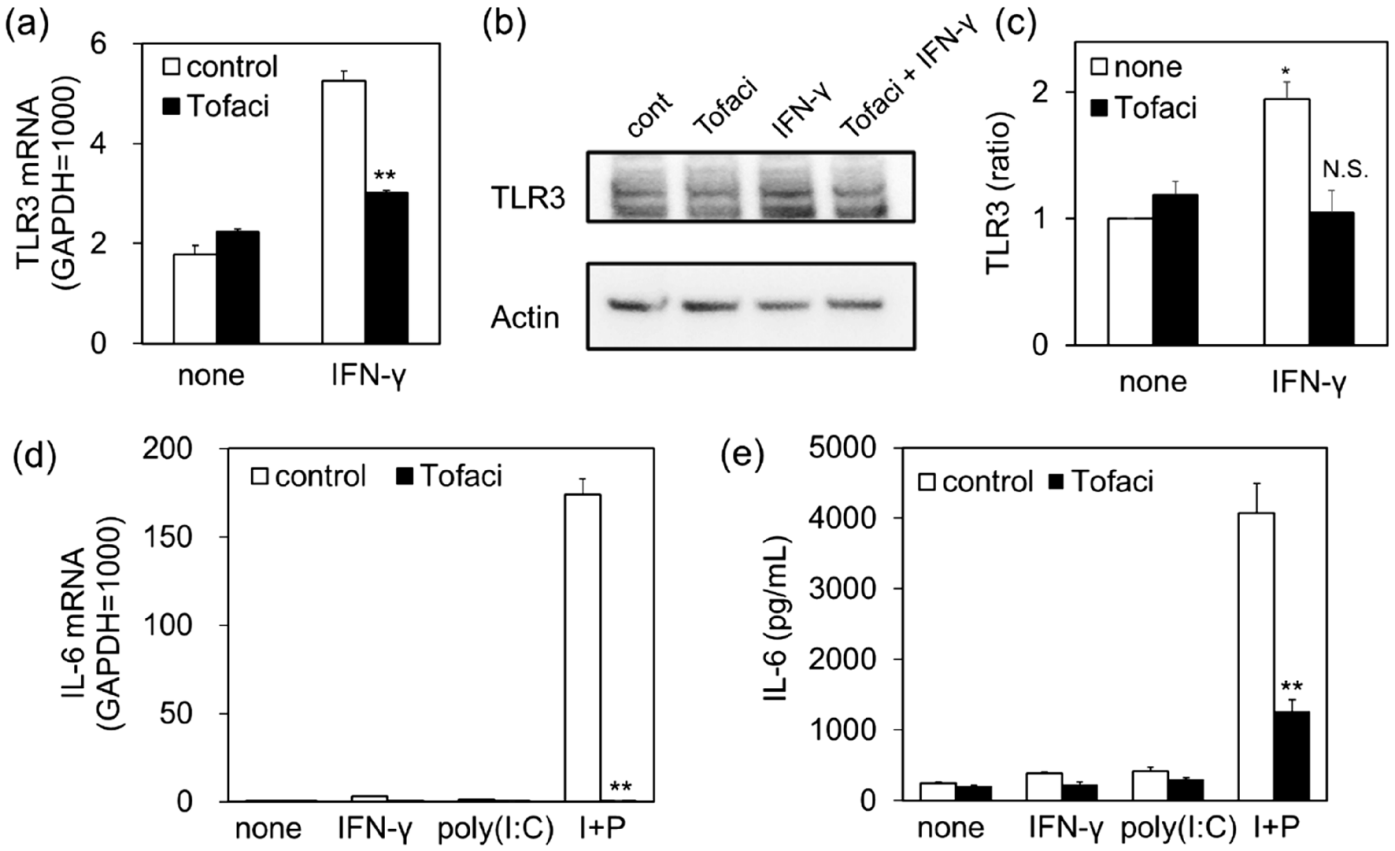
Effects of tofacitinib on the effects of IFN-γ-induced increase in TLR3 mRNA and protein, and poly(I:C)-induced IL-6 production in NCI-H292 cells. (**a**) Cells were stimulated with IFN-γ (10 ng/mL) in the absence or presence of tofacitinib (1 µM) for 18 h. TLR3 mRNA expression was examined by quantitative RT-PCR. Data were normalized to GAPDH mRNA levels. Values are shown as mean ± SEM (n = 3). **P < 0.01 versus the response to IFN-γ-induced effects. (**b**) Cell lysate was subjected to western blot to detect TLR3 protein. The blots were cut prior to incubation with antibodies. Result shown is representative image from three different experiments. (**c**) The densitometry value of protein bands of TLR3 are shown as relative intensities with that of actin. Values are shown as mean ± SEM (n = 3). *P < 0.05 versus the response to control (none). N.S. not significant versus tofacitinib alone. (**d**) Cells were pretreated with IFN-γ (10 ng/mL) for 12 h in the presence (black column) or absence (open column) of tofacitinib (1 µM), followed by stimulation with vehicle (none) or poly(I:C) (1 µg/mL) for 3 h. I + P indicates stimulation with poly(I:C) in IFN-γ-treated cells. IL-6 mRNA expression was examined by quantitative RT-PCR. Data were normalized to GAPDH mRNA levels. Values are shown as mean ± SEM (n = 3). **P < 0.01 versus the response to control. (**e**) Cells were stimulated as in (d) for 6 h. The levels of IL-6 in the reaction medium were measured by ELISA. Values are shown as mean ± SEM (n = 3). **P < 0.01 versus the response to control.

IFN-γ binds to heterodimeric receptors consisting of IFN-γ receptor (IFNGR) 1 and IFNGR2 and activates the downstream JAK-signal transducer and activator of transcription 1 (STAT1) signaling pathway to induce the expression of IFN-stimulated genes^21^. IFN-γ-induced TLR3 gene expression in NCI-H292 cells was suppressed by JAK inhibitor tofacitinib (Fig. 5a). Tofacitinib also inhibited IFN-γ-induced TLR3 protein increase (Fig. 5b,c). Tofacitinib consistently inhibited poly(I:C)-induced IL-6 mRNA elevation (Fig. 5d) and IL-6 secretion (Fig. 5e) in IFN-γ-primed NCI-H292 cells.

### Involvement of activation of NF-κB as a downstream signal of poly(I:C) stimulation

**Figure 6:**
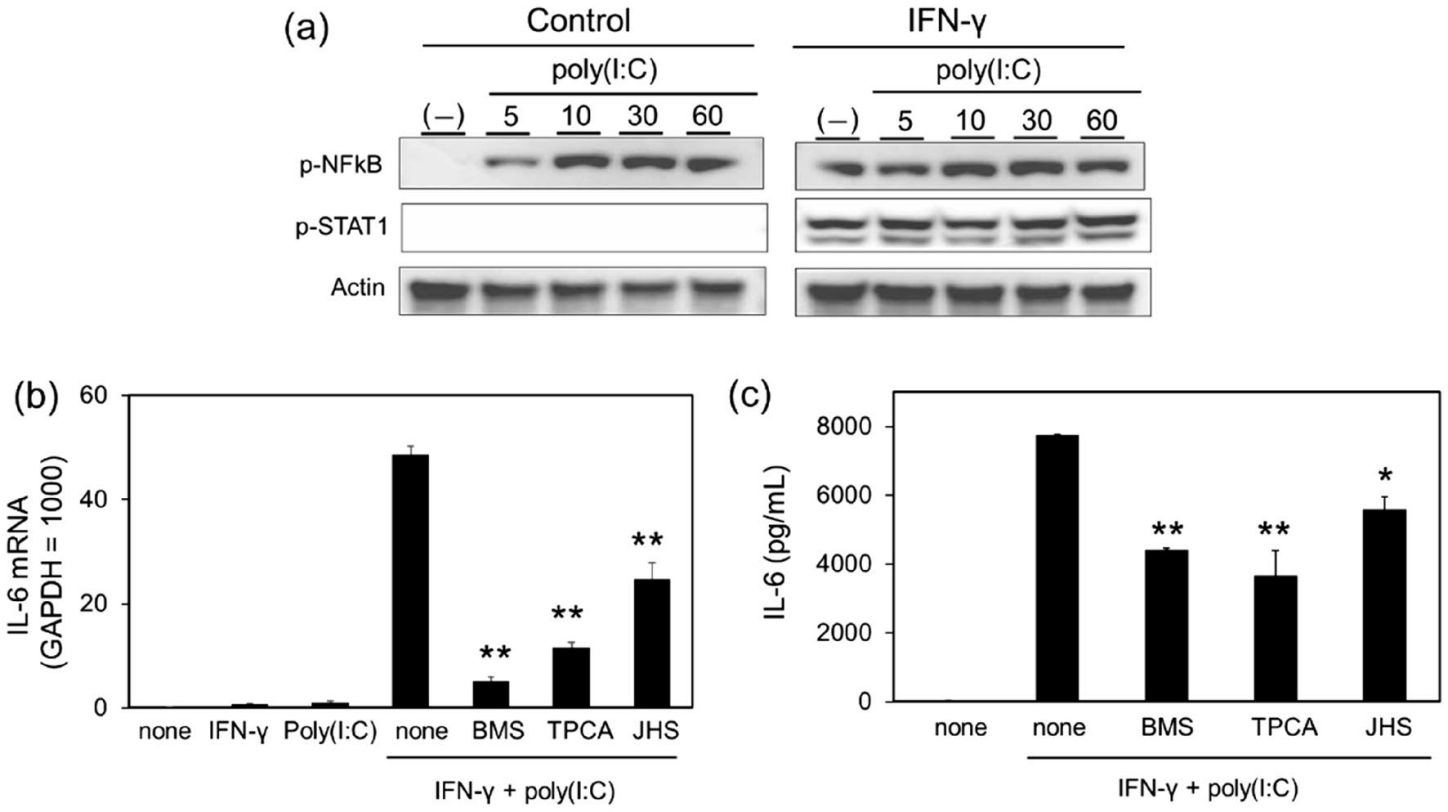
Effects of NF-κB inhibitors on poly(I:C)-induced IL-6 production in IFN-γ-treated NCI-H292 cells. (**a**) Cells were stimulated with poly(I:C) (1 µg/mL) for 5, 10, 30, and 60 min with or without of IFN-γ (10 ng/mL) pretreatment for 12 h. Cell lysates were subjected to western blot analysis for phospho-NF-κB (p-NF-κB), phospho-STAT1 (p-STAT1) and actin. Result shown is representative image. (**b**,**c**) Cells were pretreated with IFN-γ (10 ng/mL) for 12 h, followed by stimulation with poly(I:C) (1 µg/mL) in the absence or presence of BMS345541 (5 μM), TPCA1 (10 μM), and JHS-23 (10 μM) for 3 h for mRNA (**b**) and 6 h for ELISA (**c**). IL-6 mRNA expression was examined by quantitative RT-PCR. Data were normalized to GAPDH mRNA levels. The levels of IL-6 in the reaction medium were measured by ELISA. Values are shown as mean ± SEM (n = 3). *P < 0.05, **P < 0.01 versus the response to IFN-γ and poly(I:C).

Transcription factor NF-κB is activated downstream of TLR3. Stimulation of NCI-H292 cells with poly(I:C) resulted in increasing phosphorylation of NF-κB p65 subunit, an indicator of NF-κB activation, in a time dependent manner (Fig. 6a). Poly(I:C) had no effect on the phosphorylation of STAT1 (Fig. 6a). In contrast, IFN-γ stimulated STAT1 phosphorylation and increased the basal level of NF-κB p65 phosphorylation, but not largely affected the poly(I:C)-induced NF-κB p65 phosphorylation in NCI-H292 cells (Fig. 6a). Poly(I:C)-induced increases in IL-6 mRNA (Fig. 6b) and IL-6 secretion (Fig. 6c) in IFN-γ-treated NCI-H292 cells that were significantly inhibited by treatment with BMS345541 (5 µM) and TPCA1 (10 µM), IκB kinase inhibitors, and JHS-23 (10 µM), an inhibitor of the nuclear transfer of NF-κB.

### Histone modification of IL-6 gene locus by IFN-γ

**Figure 7:**
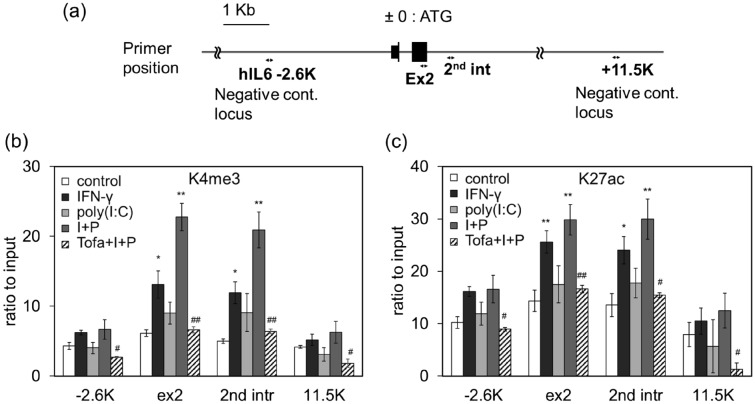
Effects of IFN-γ and poly(I:C) on modification of IL-6 gene region in NCl-H292 cells. (**a**) The diagram represents the human IL-6 locus and the position of primer sets used. NCI cells were pretreated with IFN-γ (10 ng/mL) for 12 h, followed by stimulation with poly(I:C) (1 µg/mL) for 3 h. Enrichment of histone H3K4me3 (**b**) and H3K27ac (**c**) were measured using a ChIP assay and quantitative PCR at the IL-6 locus. Data were normalized to the input DNA. *P < 0.05, **P < 0.01 versus the response to control. ^#^*P* < 0.05, ^##^*P* < 0.01 versus the response to I + P which means stimulation with IFN-γ and poly(I:C). Values are shown as the mean ± SEM. control (n = 7), IFN-γ (n = 5), poly(I:C) (n = 5), I + P (n = 5), tofaci + I + P (n = 3).

IFN-γ stimulation has been shown to evoke chromatin modifications to facilitate inflammatory cytokine gene expression in response to LPS-induced TLR4 activation in macrophages^22^. Histones H3K4me3 and H3K27ac are enhancer-specific modifications required for enhancers to activate the transcription of target genes. To investigate whether IFN-γ induced histone modifications (H3K4me3 and H3K27ac) related to IL-6 transcription, we searched the Chromatin immunoprecipitation (ChIP)-seq database of the IL-6 gene locus in various human cell lines, and selected exon2 and second intron sequences that undergo high levels of methylation and acetylation, and two regions at − 2.6 kb and + 11.5 kb as a negative control sequence (Supplementary Fig. 1 and Fig. 7a). Based on these data, we prepared primers that amplify four different sites, two transcriptionally relevant and two nearby less relevant parts of the IL-6 gene locus (Supplementary Table 1). The ChIP assay was performed using K4 trimethylated and K27 acetylated antibodies against histone H3. As shown in Fig. 7, K4 trimethylation (Fig. 7b) and K27 acetylation (Fig. 7c) were significantly enhanced by IFN-γ in the exon2 and second intron regions of the IL-6 gene locus, but not in the negative control regions. Poly(I:C) alone had little effect, and only slightly increase the K4 trimethylation but not K27 acetylation in the IFN-γ priming cells. IFN-γ-induced enhancement of K4 trimethylation (Fig. 7b) and K27 acetylation (Fig. 7c) in NCI-H292 cells was suppressed by tofacitinib. These results suggest that IL-6 gene locus presented chromatin remodeling by IFN-γ via the JAK pathway.

### Effects of IFN-γ on IL-6 production in poly(I:C)-induced acute pulmonary inflammation model mice

**Figure 8:**
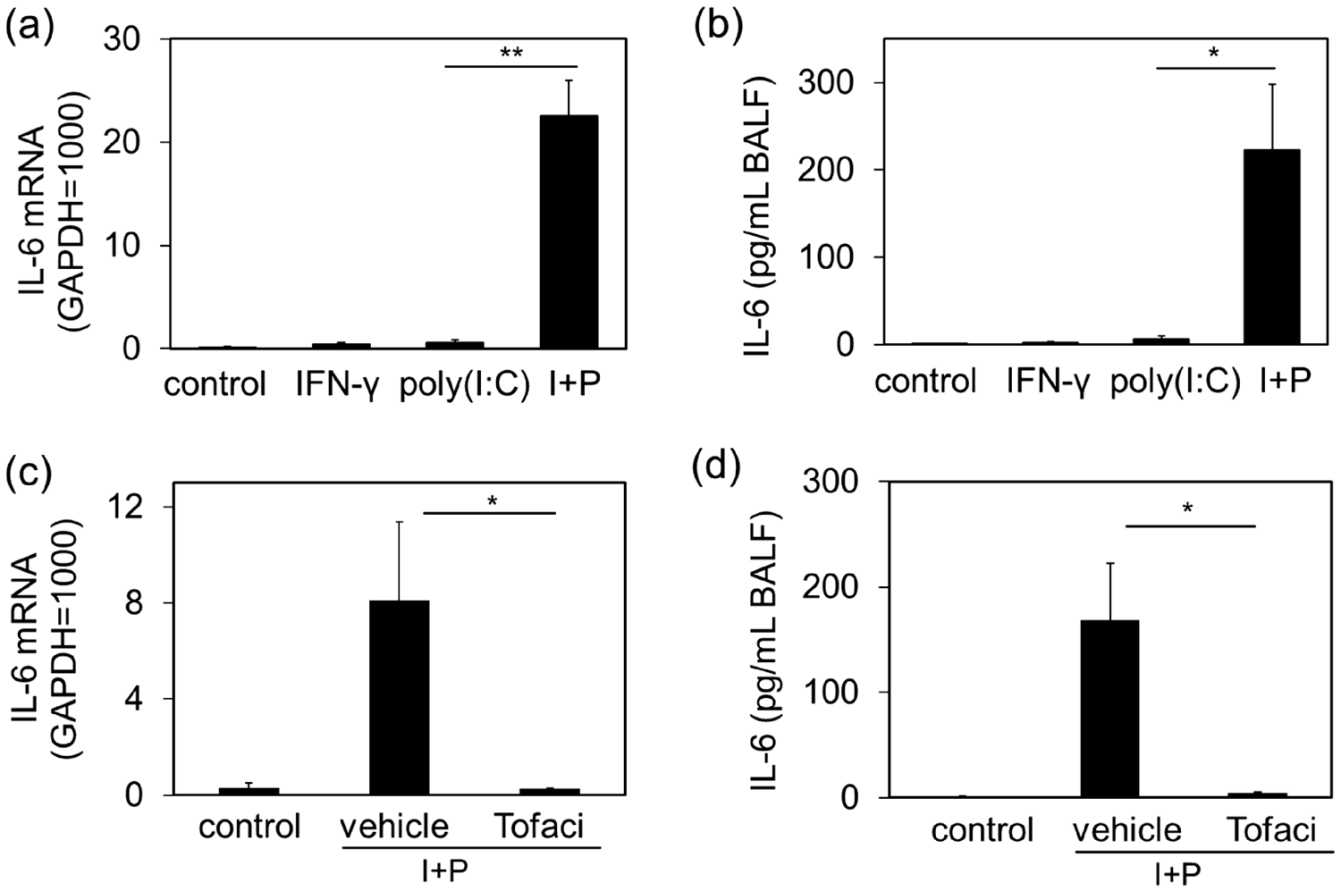
Priming effect of IFN-γ on poly(I:C)-induced IL-6 production in vivo. Mice were administered with IFN-γ (2 µg/kg) to the respiratory tract by nasal drops. After 6 h, poly(I:C) (2 mg/kg) was administered via nasal drops into the respiratory tract. At 6 h post-administration, BALF and lung tissues were collected from the mice. (**a**) IL-6 mRNA levels in lung tissues were examined using quantitative RT-PCR. Data were normalized to GAPDH mRNA levels. (**b**) IL-6 levels in BALF were measured using ELISA. Values are shown as the mean ± SEM (n = 4–5). *P < 0.05, **P < 0.01 versus the response to poly(I:C). (**c**,**d**) Same experiments were performed with mice pretreated with tofacitinib (Tofaci, 10 mg/kg)**.** Values are shown as the mean ± SEM. (n = 4–6). *P < 0.05 versus the response to vehicle.

Finally, we examined whether the priming effect of IFN-γ on poly(I:C)-induced IL-6 production observed in NCI-H292 cells occurred in vivo using a mouse model of acute pneumonia generated by intratracheal administration of poly(I:C). Only slight increase in IL-6 mRNA of lung tissue (Fig. 8a) and IL-6 protein in the bronchoalveolar lavage fluid (BALF) (Fig. 8b) was observed in mice administered with 2 mg/kg of poly(I:C). IFN-γ (2 µg/kg) alone also had little effect on IL-6 mRNA and protein levels in lung tissues (Fig. 8a,b). However, STAT1 phosphorylation (Supplemental Fig. 2Sa,b) and TLR3 mRNA levels (Supplemental Fig. 2Sc) in lung tissue were significantly increased after 6 h treatment with IFN-γ. Application of poly(I:C) to such IFN-γ pretreated mice markedly increased IL-6 mRNA levels in lung tissue (Fig. 8a), accompanied by a significant increase in IL-6 levels in BALF. Furthermore tofacitinib (10 mg/kg, i.p.) inhibited the IL-6 mRNA elevation in lung tissue (Fig. 8c) and IL-6 accumulation in BALF (Fig. 8d) induced by poly (I:C) in IFN-γ-pretreated mice.

## Discussion

In this study, we demonstrated that, in human bronchial epithelial NCI-H292 cells, IFN-γ treatment dramatically enhanced IL-6 production stimulated by poly(I:C), a double-stranded RNA analog that accumulates in virus-infected cells. When NCI-H292 cells were stimulated by IFN-γ or poly(I:C) alone, only a slight increase in IL-6 gene expression was observed; however, when cells were pretreated with IFN-γ, poly(I:C) significantly enhanced IL-6 production depending on the IFN-γ-preincubation time. These reactions were observed not only in NCI H292 cells, but also in other human respiratory epithelial cells, including alveolar epithelial A549 cells and primary cultured human normal bronchial epithelial cells HNBE, suggesting that this reaction may occur in respiratory epithelial cells.

Previous studies reported that bronchial epithelial cells produce various inflammatory cytokines, including IL-6, in response to poly(I:C)^12,23–25^. However, to the best of our knowledge, this is the first study to demonstrate that IFN-γ priming markedly enhances poly(I:C)-induced IL-6 production. Alveolar macrophages and infiltrating immune cells are thought to be the major source of IL-6^26^, but our finding that poly(I:C)-induced IL-6 production in bronchial epithelial cells is enhanced by IFN-γ priming may be important for understanding the mechanism of cytokine overproduction associated with the exacerbation of viral infections, such as SARS-Cov-2 and influenza.

Poly(I:C) is a synthetic analog of viral dsRNA and a well-characterized ligand of TLR3^13^. The results of the present study suggest that the marked enhancement of poly(I:C)-induced IL-6 production by IFN-γ is accompanied by the upregulation of TLR3. That is, IFN-γ increased TLR3 mRNA expression and protein levels in NCI-H292 cells. Furthermore, knockdown of TLR3 with siRNA effectively abrogated the enhancement by IFN-γ of poly(I:C)-induced IL-6 production.

Interestingly, upregulation of TLR3 expression in NCI-H292 cells was not observed with type I IFN, TNF-α, IL-1β, the TLR4 ligand LPS, or histamine, which is consistent with finding that the enhancement of IL-6 production was not significant with such cytokines. In addition to TLR3, virus-derived dsRNAs are recognized by various innate immune sensors including retinoic acid-inducible gene I (RIG-I), melanoma differentiation-associated gene 5 (MDA-5), and dsRNA-dependent protein kinase (PKR)^27^. Matsukura et al. reported that the poly(I:C)-induced inflammatory cytokine production in bronchial epithelial cells was inhibited by siRNA targeting TLR3, but not by RIG-I, MDA-5, or PKR^24^. The role of RNA sensors other than TLR3 in the response to the combination of poly(I:C) and IFN-γ needs to be clarified in future studies.

IFN-γ is a key endogenous activator of macrophages and potentiates the actions of many TLRs and inflammatory cytokines^19^. The IFN-γ receptor is composed of the ligand-binding subunit IFNGR1 and signaling subunit IFNGR2. Functional IFN-γ receptors are expressed not only in immune cells but also in a variety of cell types, including epithelial cells^19^. Binding of IFN-γ to IFNGR1 forms a functional complex with IFNGR2 to activate receptor-associated tyrosine kinases JAK1 and JAK2, which in turn phosphorylate STAT1 to form homodimers, translocate to the nucleus, and activate the transcription of target genes^28^. Enhancement of poly(I:C)-induced IL-6 production by IFN-γ was strongly suppressed by the JAK inhibitor tofacitinib, suggesting the importance of STAT1-mediated regulation. IFN-α also phosphorylates STAT1, but forms a heterotrimer with phosphorylated STAT2 and IRF9^29^. This difference may account for the differential effects of IFN-α and IFN-γ on TLR3 induction.

Expression of the IL-6 gene is under the control of many different transcription factors such as NF-κB, SP (specificity protein) 1, AP-1 (activator-protein-1), CREB (cyclic AMP-responsive element-binding protein), and C/EBP (CCAAT-enhancer-binding protein^30^. The results of this study show that poly(I:C) activated the NF-κB pathway, but alone had a weak effect on IL-6 mRNA expression. However, stimulation of the IFN-γ receptor activates JAK-STAT signaling pathway and phosphorylated STAT1 homodimer is translocated to nuclei to associate with IFN-γ activation site (GAS) element on DNA to regulate gene expression^31^. Although the human IL-6 promoter does not contain GAS regulatory elements, STATs recruit histone acetyltransferases and chromatin remodeling enzymes^28^. These enzymes are known to induce chromatin remodeling and indirectly enhance gene expression by transcription factors such as NF-κB^18^. The present results demonstrate that the priming effect of IFN-γ on poly(I:C)-induced IL-6 production may occur via a similar mechanism. In fact, ChIP assay showed that IFN-γ stimulates H3K27ac and H3K4me3 at the IL-6 gene locus in NCI H292 cells. Unlike IFN-γ, poly(I:C) induced no such histone modifications by itself, and only slightly increased IFN-γ–induced histone modifications, which was much less than the synergistic effect observed in enhancing IL-6 production. In addition, tofacitinib completely inhibited IFN-γ-induced histone methylation and acetylation at the IL-6 gene locus. These results suggest that IFN-γ induces chromatin remodeling of the IL6 gene via the JAK-STAT1 signaling pathway and promotes IL-6 gene expression induced by the poly(I:C)-TLR3-NF-κB pathway.

Finally, we demonstrated that the enhanced poly(I:C)-induced IL-6 production by IFN-γ observed in human bronchial epithelial cells could be reproduced in a mouse model of poly(I:C)-induced acute pneumonia. Similar to the results obtained with cell lines in vitro, IL-6 gene expression in lung tissue and IL-6 levels in BALF in a poly(I:C)-induced acute pneumonia were significantly enhanced by IFN-γ. We confirmed that IFN-γ treatment increased phosphorylated STAT1 levels in lung tissue (Supplemental Fig. S2). The enhancing effect of IFN-γ on poly(I:C)-induced IL-6 production was inhibited by treatment of mice with tofacitinib. Although the involvement of cells other than bronchial epithelial cells as the cannot be excluded from the results obtained in this mouse model, bronchial epithelial cells may also be involved in the accumulation of IL-6 in the lungs. To address this issue, further investigations using primary bronchial epithelial cells from mice and bronchial epithelium-specific TLR3 knockout mice are required. In addition, it is also important to immunohistochemically analyze the lung tissues of pathological model mice treated with IFN-γ and poly(I:C).

The augmentation of poly(I:C)-induced IL-6 production by IFN-γ shown in this study may serve as a model for excess inflammatory cytokine production in the lungs of severe COVID19 patients. In fact, IFN-γ is reported to be an independent risk factor associated with mortality in patients with moderate and severe COVID-19 infection^21^. Furthermore, type 2 diabetes and coronary artery disease are known to be risk factors for exacerbation in COVID19 patients^3^, and it has been reported that elevated IFN-γ levels are present in the background of these diseases^32,33^. Considering these reports, the prophylactic administration of JAK inhibitors, such as tofacitinib, may be a useful strategy to prevent the exacerbation of various viral infections, including COVID-19. In fact, clinical trials have been conducted about efficacy and safety of JAK inhibitors including tofacitinib for COVID-19^11,34^.

Taken together, our findings indicate that IFN-γ upregulates the expression of IL-6 in response to synthetic dsRNAs poly(I:C) via TLR3-NF-κB signaling in bronchial epithelial cells. Although further research using viruses and clinical studies in patients are needed, the present results may expand our understanding of the mechanisms underlying bronchial viral infection and subsequent bronchial inflammation.

## Materials and methods

### Materials

TLR3 agonist poly(I:C) was purchased from Tocris Bioscience (Bristol, UK). Recombinant Human IFN-γ was purchased from PeproTech (Tokyo, Japan). Anti-TLR3 (#6961), anti-phospho-NF-κB (#3033), anti-phospho-STAT1 (#9167), and horseradish peroxidase (HRP)-linked anti-rabbit IgG (#7074) antibodies were purchased from Cell Signaling Technology (Danvers, MA, USA). Anti-Actin antibody (sc1616-R) was purchased from Santa Cruz Biotechnology (Santa Cruz, CA, USA). Anti-K4me3 (ab8580) and anti-K27ac (ab4729) antibodies were purchased from Abcam (Cambridge, UK). Normal Rabbit IgG (12–370) was purchased from Upstate Biotechnology (Waltham, MA, USA). BMS345541 (IKK inhibitor) and JSH-23 (NF-κB transcriptional activity inhibitor) were from Cayman Chemical (Ann Arbor, MI, USA). The human IL-6 enzyme-linked immuno-sorbent assay (ELISA) kit was purchased from Thermo Fisher Scientific (Waltham, MA, USA). All other chemicals used were of reagent grade or the highest quality available.

### Cell culture

Human bronchial epithelial (NCI-H292) and human alveolar epithelial (A549) cells were obtained from the ATCC RIKEN Bioresource Center (Tukuba, Japan) and grown in RPMI1640 growth medium and Dulbecco’s modified Eagle’s medium (DMEM) supplemented with 5% (v/v) heat-inactivated fetal bovine serum, 100 U/mL penicillin, and 100 μg/mL streptomycin. Human primary bronchial epithelial (NHBE-Bronchial Epi without RA, CC-2541) cells were obtained from Lonza (Tokyo, Japan) and grown using a Bronchial Epithelial Cell Growth Medium Bullet Kit (Lonza, Tokyo, Japan) (passage number 3–5). These cells were maintained in 100 mm dishes and incubated at 37 °C in a humidified atmosphere of 95% air and 5% CO_2_.

### siRNA-mediated knockdown

NCI cells were seeded in 12-well plates. After 24 h, NCI cells were transfected with 50 pmol/well of human TLR3 gene-specific Silencer® Select siRNA (siRNA ID:s235; Invitrogen, Tokyo, Japan) or Silencer® Select Negative Control siRNA using Lipofectamine™ RNAiMAX diluted in Opti-MEM, according to the manufacturer’s instructions. The culture medium was replaced 1 d after siRNA treatment. After another 1 d of culture, the NCI cells were used for mRNA and cytokine measurements.

### Quantitative reverse transcription-PCR

Cells were seeded in a 12-well plate and grown in RPMI 1640 growth medium and then stimulated under various conditions for different periods at 37 °C. Total RNA was isolated using the NucleoSpin RNA kit (Macherey–Nagel, Düren, Germany). First-strand cDNA was synthesized using Moloney murine leukemia virus reverse transcriptase and 6-mer random primers (Takara Bio, Shiga, Japan). Quantitative reverse transcription-PCR was performed using an Mx3000P real-time PCR system (Agilent Technologies, Santa Clara, CA, USA) with TB Green Premix Ex Taq II (Tli RNase H Plus; Takara Bio, Shiga, Japan). The results were normalized to the expression of glyceraldehyde-3-phosphate dehydrogenase (*GAPDH*). The primer sequences used for real-time PCR are listed in supplementary Table 1.

### Measurement of IL-6 secretion

The cells were seeded in a 48-well plate and grown to confluence in RPMI 1640 growth medium. Cells were washed with RPMI medium and then stimulated under various conditions for different periods at 37 °C. IL-6 protein in the culture medium was assayed using a human IL-6 enzyme-linked immunosorbent assay (ELISA) kit (Thermo Fisher Scientific, Waltham, MA, USA), according to the manufacturer’s instructions.

### Western blotting

Cells were seeded in a 12-well plate and grown in RPMI 1640 growth medium and then stimulated under various conditions for different periods at 37 °C. The reactions were terminated by the addition of Laemmli sample buffer. The lysates were separated by 10% sodium dodecyl sulfate–polyacrylamide gel electrophoresis (SDS-PAGE) and transferred onto Immobilon-P polyvinylidene fluoride membranes. Membranes were blocked with 5% nonfat milk for 1 h and incubated with primary antibodies for 12 h at 4 °C and secondary antibodies for 2 h at room temperature. The antibodies were diluted as follows: anti-TLR3 (1:2000), anti-phospho-NF-κB (1:1000), anti-phospho-STAT1 (1:1000), anti-Actin (1:1000), and horseradish peroxidase (HRP)-linked anti-rabbit IgG (1:10,000). Immunoreactive proteins were detected with Immobilon Western chemiluminescent HRP substrate (Merck Millipore, Tokyo, Japan) using an Image Reader LAS-3000 (FUJIFILM, Tokyo, Japan).

### Chromatin Immunoprecipitation (ChIP)

The ChIP assay was performed as previously described with slightly modification^35–37^. Briefly, a total of 5 × 10^6^ NCI-H292 cells per immunoprecipitation (IP) were fixed with 1% formaldehyde at room temperature for 10 min and lysed. The samples were sonicated to shear the DNA using a focused ultrasonicator M220 (Covaris) with duty factor 5%, peak incident power 75 W, and 200 cycles per burst for 300 s. The solubilized chromatin fraction was incubated with the primary antibodies overnight, which were prebound to Dynabeads Protein A (Invitrogen, Carlsbad, CA, USA). The primary antibodies used for the ChIP assay were anti-H3K4me3 (ab8580; Abcam), anti-H3K27ac (ab4729; Abcam), and normal Rabbit IgG (12–370; Upstate Biotechnology) respectively. The DNA purified from the ChIP samples was decrosslinked, purified with Chelex, and analyzed using an Mx3000P real-time PCR system with TB Green Premix Ex Taq II (Tli RNase H Plus) (Takara Bio). The results obtained from IP were normalized with the results from input DNA without IP. The PCR primers used for the ChIP assays are listed in supplementary Table 1.

### Animals

All animal experimental protocols were approved by the Animal Research Committee of Takasaki University of Health and Welfare (approval no. 2033) and conducted according to the Animal Experiment Regulations of Takasaki University of Health and Welfare. The study was conducted in compliance with the Animal Research Reporting of In Vivo Experiments (ARRIVE) guidelines. C57BL/6 mice (9–12 weeks old) were obtained from SLC Japan (Hamamatsu, Japan). Mice were maintained under specific pathogen-free conditions with a 12-h light–dark cycle and free access to feed and water at room temperature of 22 ± 2 °C.

### Acute bronchial inflammation model in mice

Mice were anesthetized with isoflurane and intratracheally administered poly(I:C) (2 mg/kg) or vehicle. IFN-γ (2 µg/kg) was administered intratracheally 6 h before poly(I:C) administration. Tofacitinib (10 mg/kg) was administered intraperitoneally 0.5 h before IFN-γ. 6 h after poly(I:C) administration, the mice were euthanized by overanesthesia with pentobarbital. The bronchoalveolar lavage fluid (BALF) was collected from the mice by washing 3 times with 300 µL PBS via trachea. Obtained BALF was centrifuged at 3000 g for 10 min at 4 °C to remove cells and particulates and then used for ELISA. Lung tissues were collected and subjected to quantitative reverse-transcription PCR.

### Statistics

All values are expressed as the mean ± standard error of the mean (SEM). Data were analyzed using Mann–Whitney *U* tests for two-sample comparison, non-parametric ANOVA Kruskal–Wallis and Dunnett’s two-tailed test for multiple data comparison. *P* values < 0.05 were considered statistically significant.

### Supplementary Information

Supplementary Figures.

Supplementary Information.

## Data Availability

The datasets generated and/or analyzed in the current study are available from the corresponding author upon reasonable request.
