# Supplementary material for: Amplification of poly(I:C)-induced interleukin-6 production in human bronchial epithelial cells by priming with interferon-γ
Source: Sci Rep. 2023 Nov 29;13:21067. doi: 10.1038/s41598-023-48422-9 (PMC10687102; doi:10.1038/s41598-023-48422-9)

# Original Blot in Figure 3 (d)

TLR3

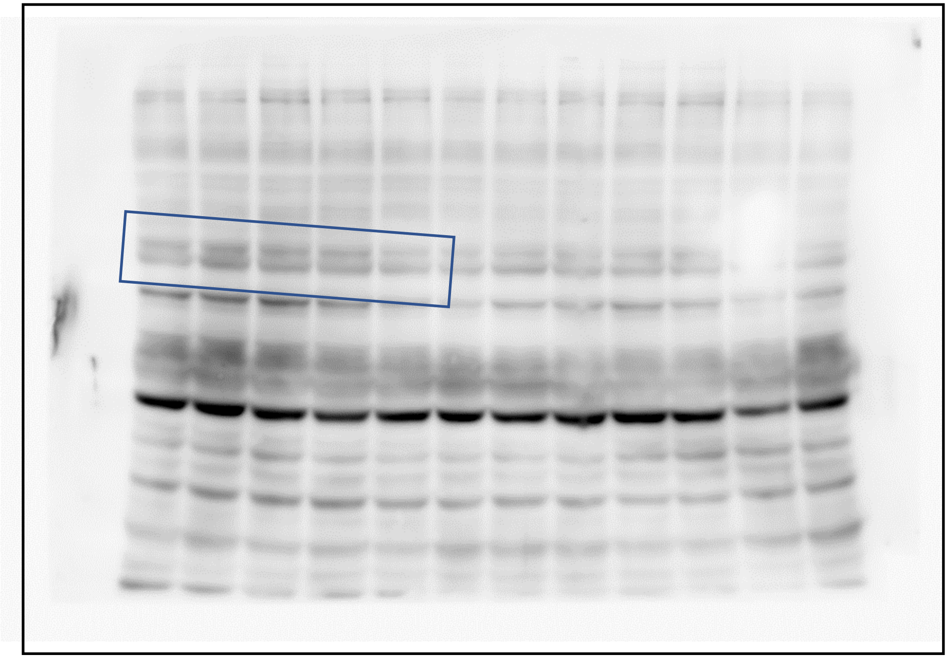

Actin

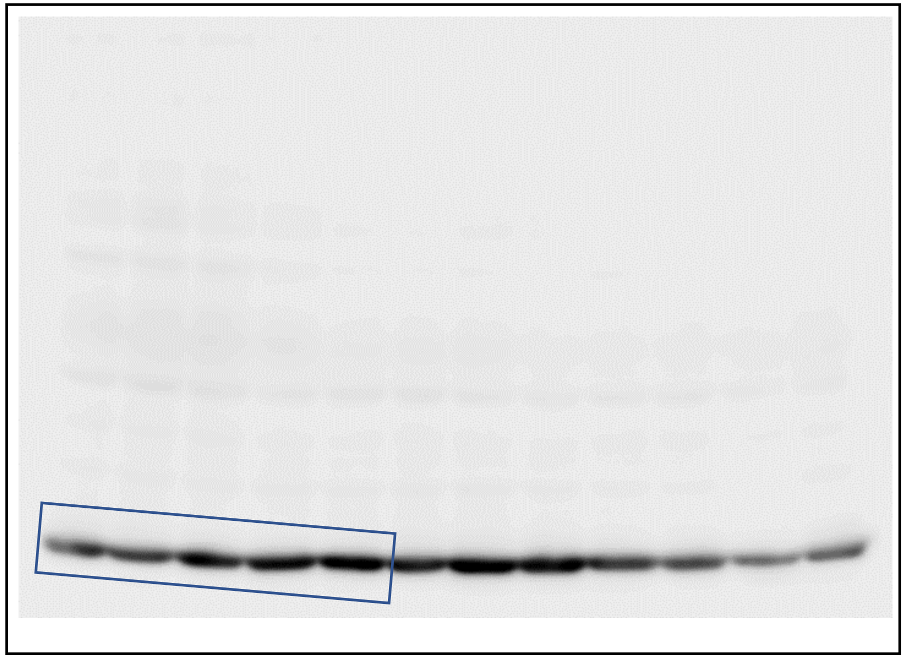

Original Blot in Figure 4 (b)

TLR3

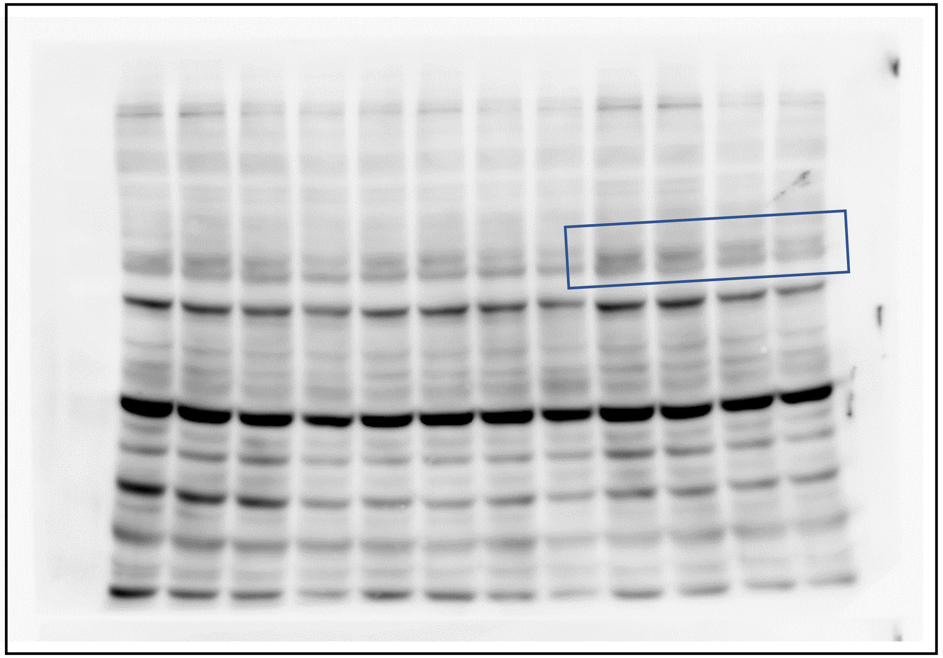

Actin

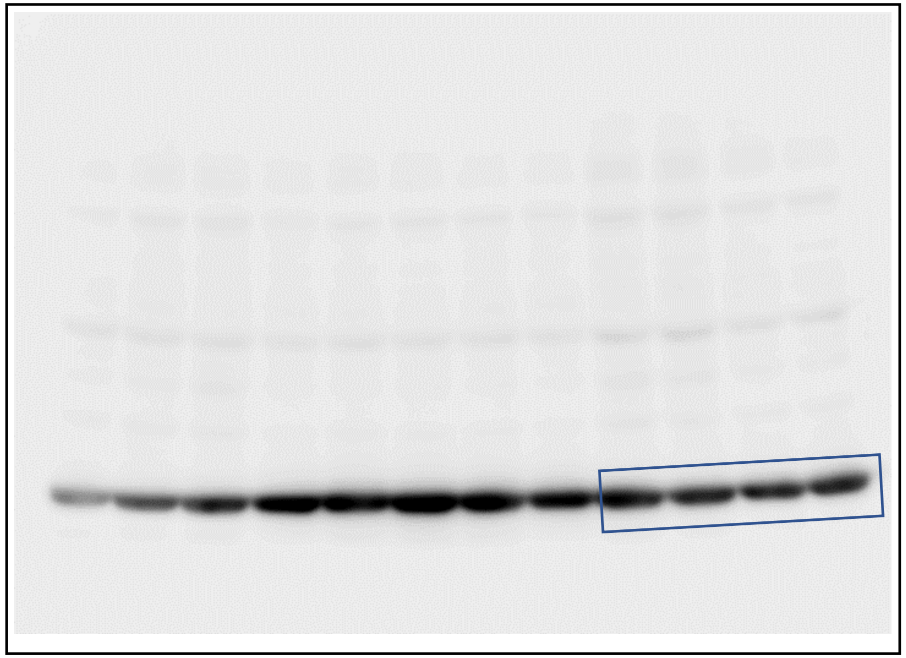

Original Blot and membrane in Figure 5 (b)

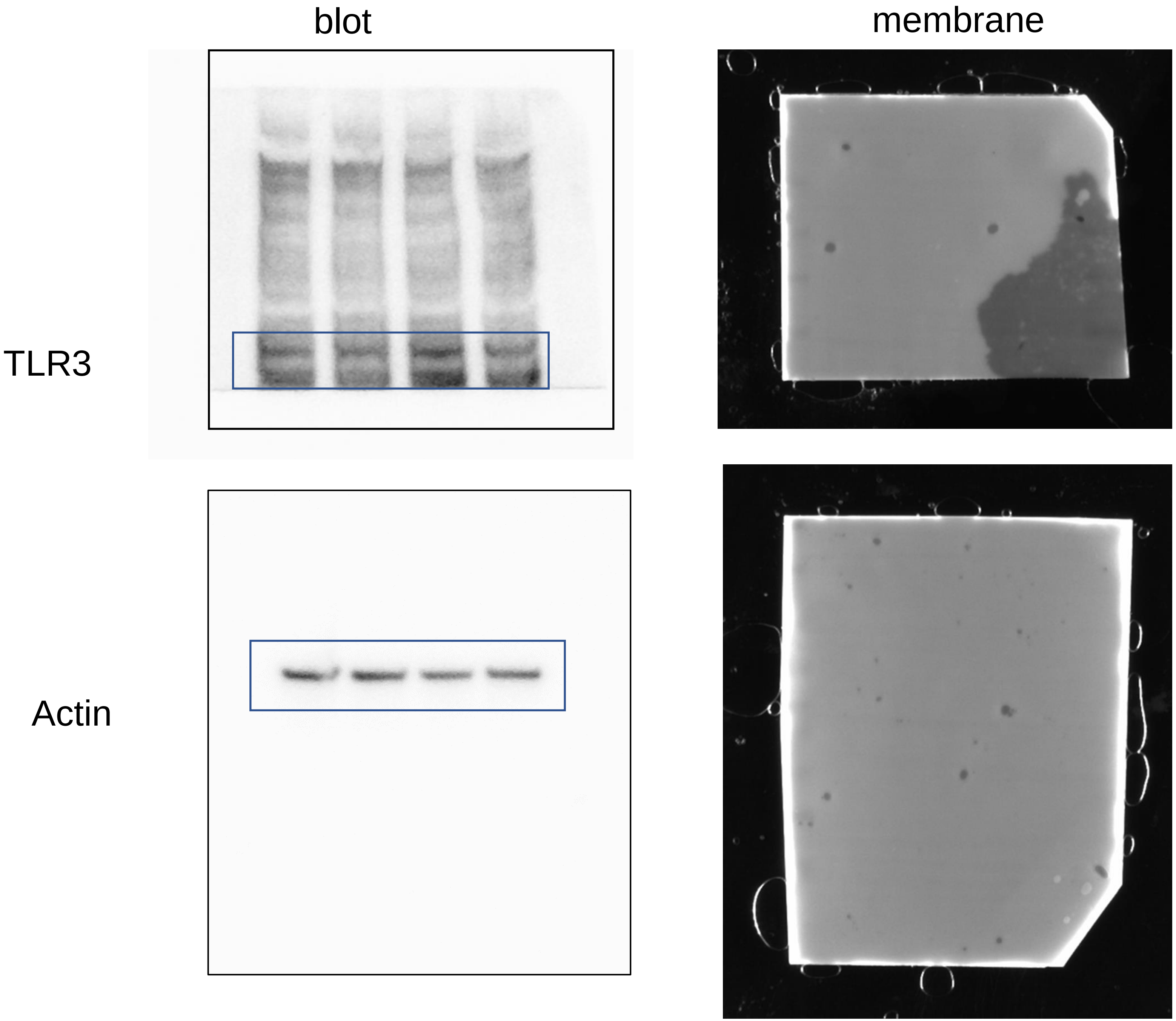


The blots were cut prior to incubation with antibodies.

# Original Blot in Fig6 (a)

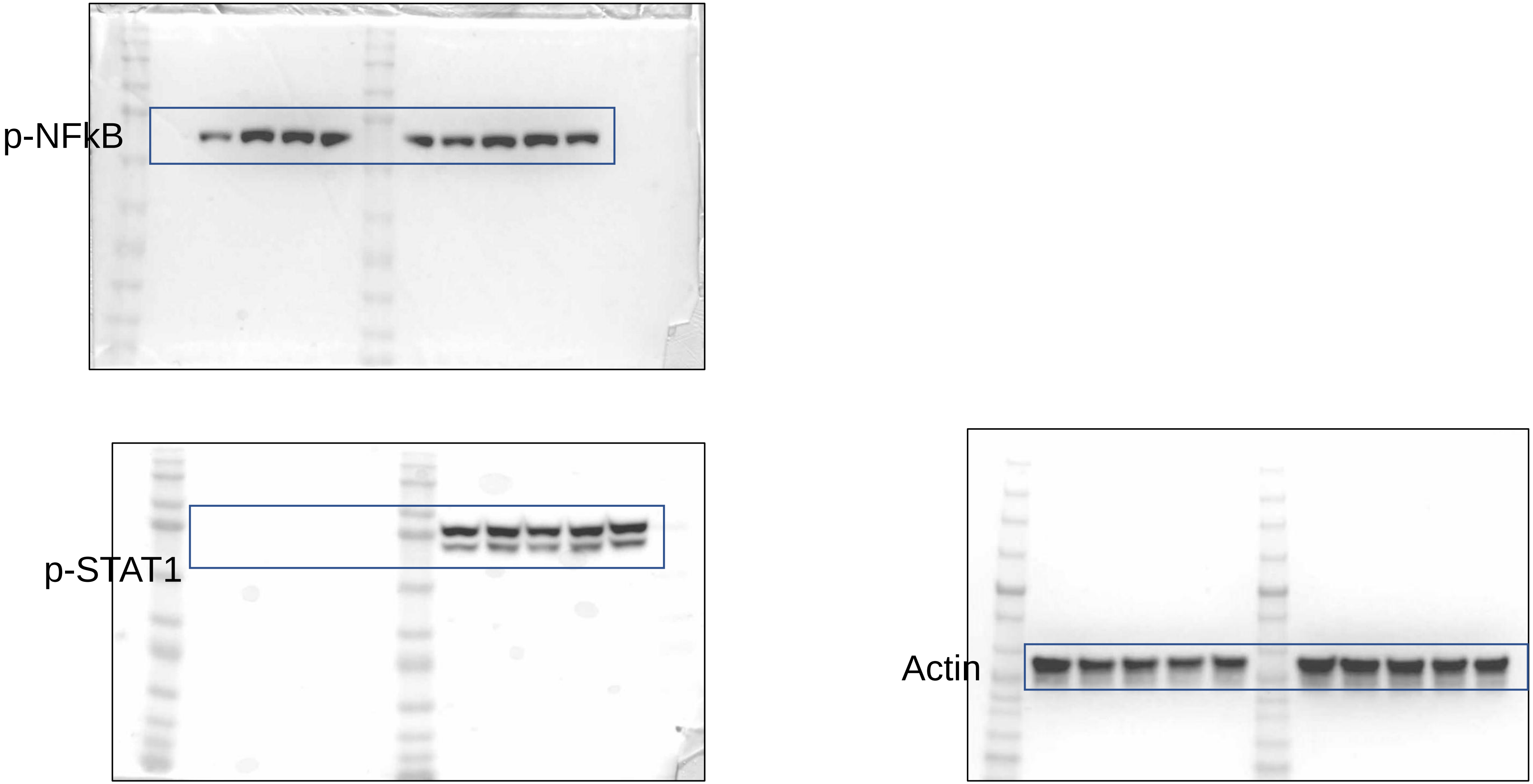

# Original Blot in Supplmental Fig S2

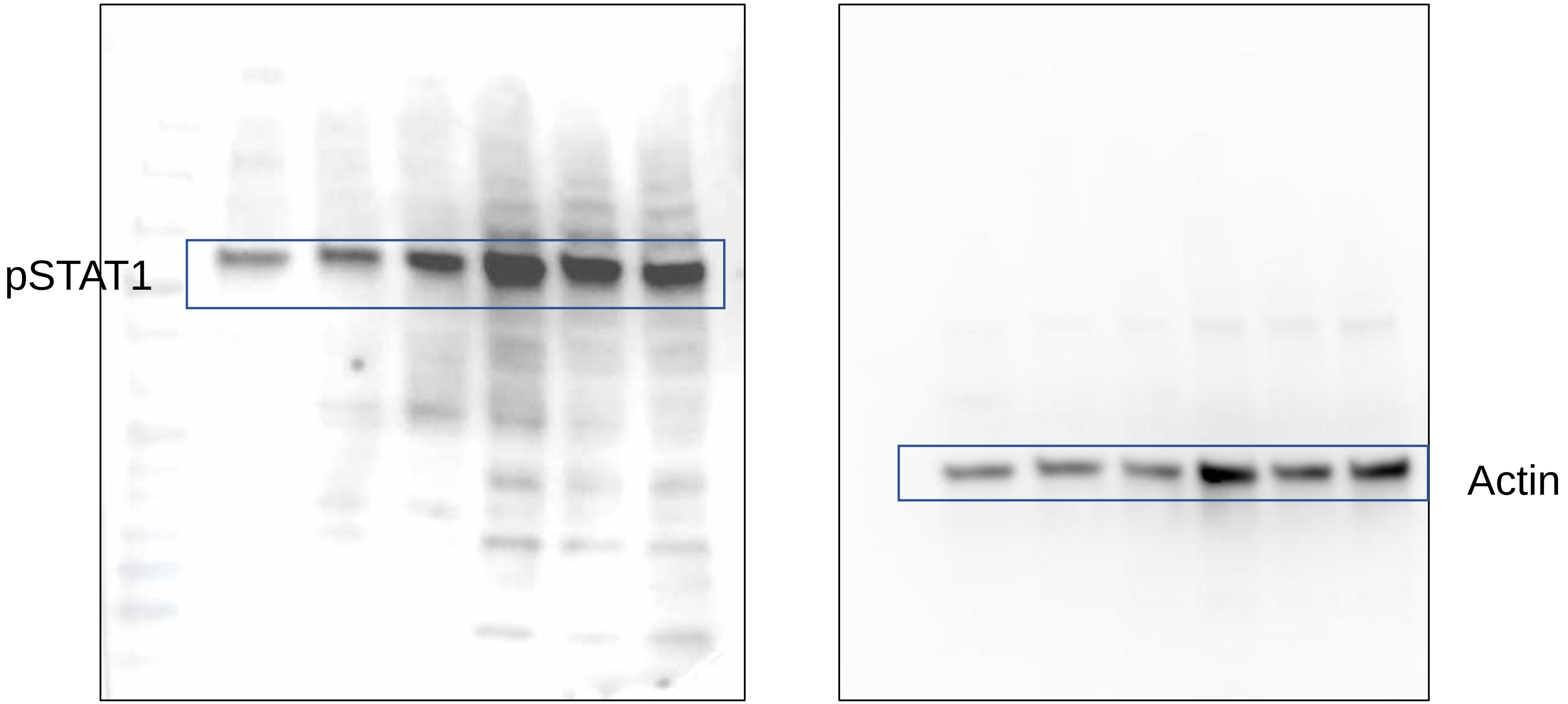

Supplement: Supplementary file 1 — Supplementary Figures. [file 41598_2023_48422_MOESM1_ESM.pdf]
