# Supplementary material for: Amplification of poly(I:C)-induced interleukin-6 production in human bronchial epithelial cells by priming with interferon-γ
Source: Sci Rep. 2023 Nov 29;13:21067. doi: 10.1038/s41598-023-48422-9 (PMC10687102; doi:10.1038/s41598-023-48422-9)
Supplement: Supplementary file 2 — Supplementary Information. [file 41598_2023_48422_MOESM2_ESM.pdf]

# Amplification of poly(I:C)-induced interleukin-6 production in human bronchial epithelial cells by priming with interferon-γ

Norikazu Okuma[1,2], Masa-aki Ito* [1], Tomoyoshi Shimizu[1], Atsuya Hasegawa[1], Shin'ya Omori[3], Kazuki Yoshida[1], Isao Matsuoka[1]

[1] Laboratory of Pharmacology, Faculty of Pharmacy, Takasaki University of Health and Welfare, Takasaki-shi, Gunma 370-0033, Japan

[2] Department of Pharmacy, Japan Community Health care Organization Gunma Chuo Hospital, Maebashi-shi, Gunma 371-0025, Japan

[3] Laboratory of Allergy, Faculty of Pharmacy, Takasaki University of Health and Welfare, Takasaki-shi, Gunma 370-0033, Japan

***Corresponding Author**: mito@takasaki-u.ac.jp

**Supplementary table 1.** Primer sequences

| name | Forward | Reverse |
|---|---|---|
| **for RT-PCR** | | |
| **GAPDH** | GAGTCAACGGATTTGGTCGT | GATCTCGCTCCTGGAAGATG |
| **IL-6** | AAATTCGGTACATCCTCGAC | CAGGAACTGGATCAGGACTT |
| **hIL-8** | TGGCAGCCTTCCTGATTTCT | TGCACTGACATCTAAGTTCTTTAGCA |
| **TNFα** | TGGCCAATGGCGTGGAGCTG | GTAGGAGACGGCGATGCGGC |
| **TLR3** | GGCTAGCAGTCATCCAACAGA | TGAAGTTGGCGGCTGGTAAT |
| **TLR4** | AGCAGTGAGGATGATGCCAG | TTAGGAACCACCTCCACGC |
| **RIG-1** | CAAATCAGAACACAGGCAGAGG | CCCATGTCTGAAGGCGTAAA |
| **MDA5** | CAACCTCGTCATTGTCAGGC | GACAGGTGAAATGTGCGTGC |
| | | |
| **for ChIP assay** | | |
| **hIL6 -2.6K** | CCACAAGCAAATTCCAGCCC | AAGGCATGTGTGTAGGGAGC |
| **hIL6 ex2** | CACAGACAGCCACTCACCTC | AGCCTACCCACCTCCTTTCT |
| **hIL6 2nd int** | TTTGGCATGAGCTGAGGGTT | TCTAGTCCTTCCAAAGCCCG |
| **hIL6 +11.5 k** | TCGGAGTTACCCAGGATGTG | TTGTCATCTCCCCACAGAAGC |

# Supplementary Figure S1.

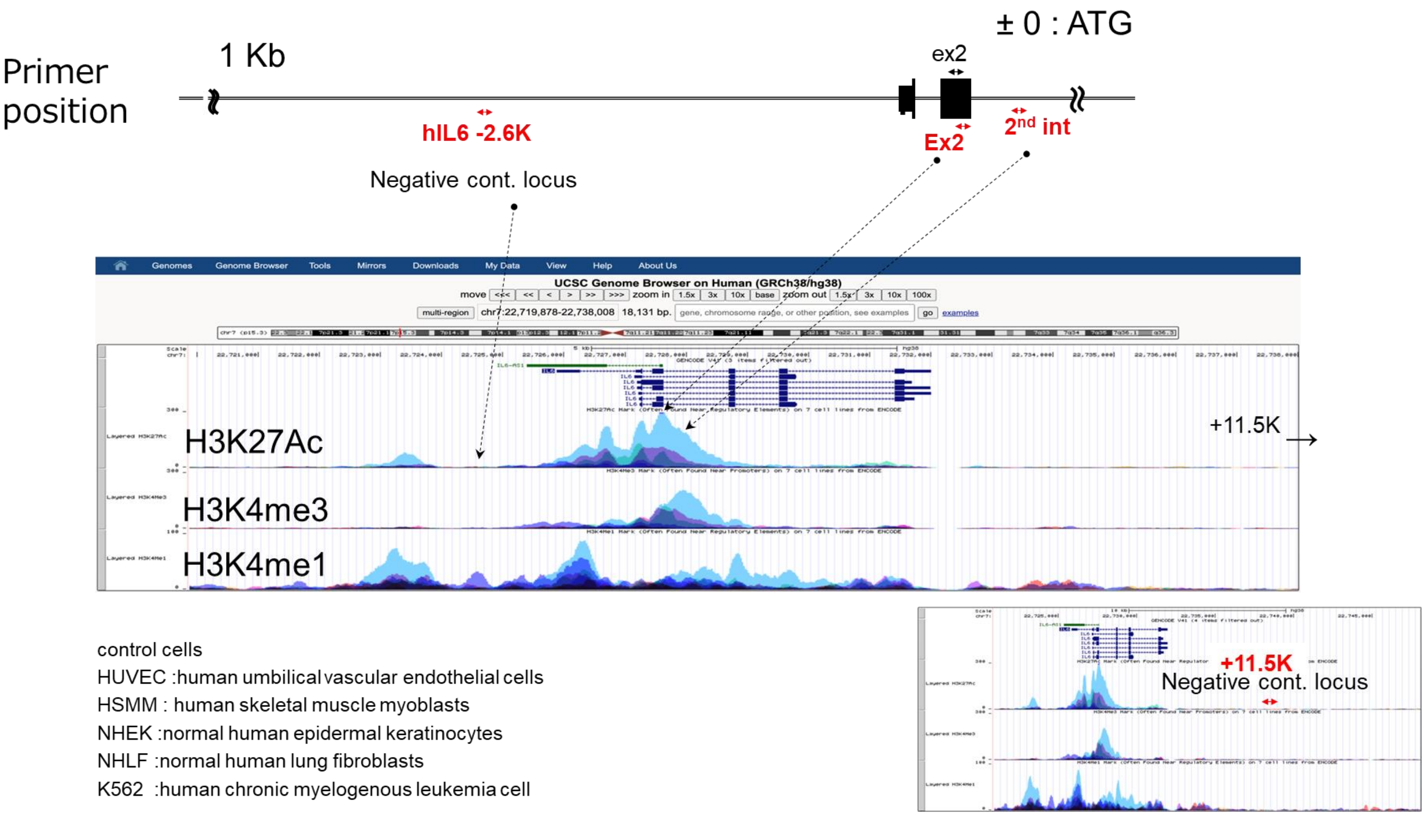


Supplemental Figure S1

Histone modification of IL-6 gene locus in various cells. Publicly available chromatin immunoprecipitation (ChIP)-seq database of the histone modification marks in the IL-6 gene locus was researched in various human cell lines such as HUVEC (human umbilical vascular endothelial cells), HSMM (human skeletal muscle myoblasts), NHEK (normal human epidermal keratinocytes), NHLF (normal human lung fibroblasts), and K562 (human chronic myelogenous leukemia cell). The peaks are visualized with IGV software and selected Ex2(exon2) and second intron (2nd int) sequences that undergo high levels of methylation and acetylation, and two regions at -2.6 kb and +11.5 kb as a negative control sequence. Based on these data, the primers that amplify several genomic regions were prepared.

# Supplementary Figure S2.

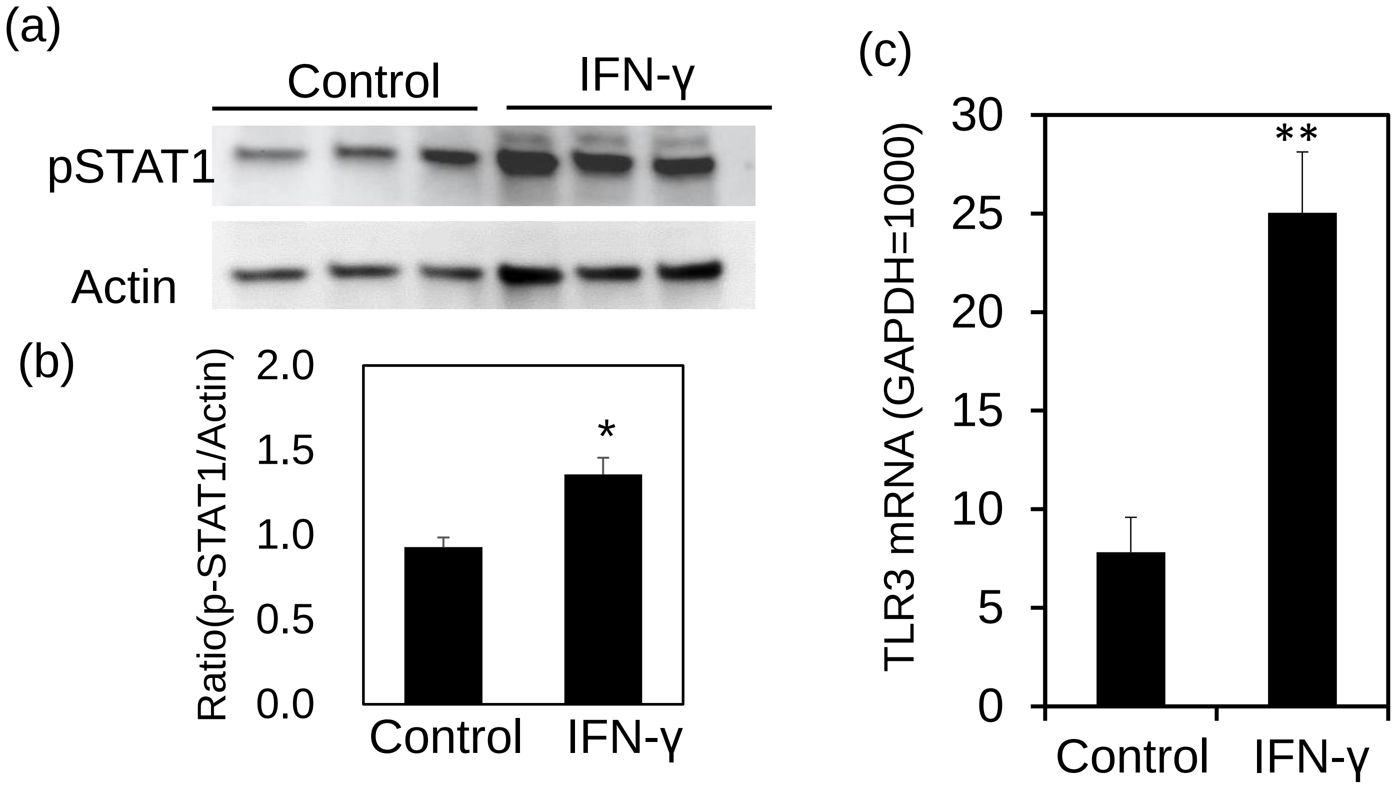


Supplemental Figure S2

IFN-γ was intranasally administered to mice, and 6 hours later, the lungs were removed, and total RNA and proteins were extracted. (a) 20 μg of protein extracted with RIPA was separated by SDS-PAGE and transferred to a PVDF membrane. The transfer membrane was incubated with anti-phosphorylated STAT1 antibody, and after washing, phosphorylated STAT1 was detected by chemiluminescence using HRP-labeled anti-rabbit antibody. (b) Membrane were then subjected to analyze actin levels using anti-actin antibody, followed by detecting by chemiluminescence using HRP-labeled anti-rabbit antibody. The densitometry value of protein bands of phospho-STAT1 （p-STAT1) are shown as relative intensities with that of actin. Data are shown as mean ± SEM (n = 3). (c) TLR3 mRNA expression was examined by quantitative RT-PCR. Data were normalized to GAPDH mRNA levels. Values are shown as mean ± SEM (n = 3). *P < 0.05,
